# Synchronization of OpenCap with Force Platforms: Validation of an Event-Based Algorithm

**DOI:** 10.3390/s26020360

**Published:** 2026-01-06

**Authors:** María Isabel Pavas Vivas, Diego Alejandro Arturo, Stefania Peñuela Arango, Jhon Alexander Quiñones-Preciado, Lessby Gomez-Salazar

**Affiliations:** 1 Program in Biomedical Sciences, School of Basic Sciences, Faculty of Health, Universidad del Valle, Cali 760032, Colombia; maria.pavas@correounivalle.edu.co (M.I.P.V.); stefania.penuela@correounivalle.edu.co (S.P.A.); 2 Program of Biomedical Engineering, Universidad Autónoma de Occidente, Cali 760037, Colombia; diego.arturo@uao.edu.co; 3 Program of Occupational Safety and Health Administration, Faculty of Virtual and Distance Education, Institución Universitaria Antonio José Camacho, Cali 760047, Colombia

**Keywords:** signal synchronization, biomechanical analysis, markerless motion capture, OpenCap, force platforms

## Abstract

Background: The integration of markerless motion capture systems such as OpenCap with force platforms expands the possibilities of biomechanical analysis in low-cost environments; however, it requires robust temporal synchronization procedures in the absence of shared hardware triggers. Objective: To develop and validate an automatic synchronization algorithm based on heel kinematic events to align OpenCap data with force platform signals during lower-limb functional exercises. Methods: Thirty normal-weight adult women (18–45 years) were evaluated while performing between 11 and 14 functional tasks (60° and 90° squats, lunges, sliding variations, and step exercises), yielding 330 motion records. Kinematics were estimated using OpenCap (four iPhone 12 cameras at 60 Hz), and kinetics were recorded using BTS P6000 force platforms synchronized with an OptiTrack system (Gold Standard). The algorithm detected heel contact from the filtered vertical coordinate and aligned this event with the initial rise in vertical ground reaction force. Validation against the Gold Standard was performed in 20 squat repetitions (10 at 60° and 10 at 90°) using Pearson correlation, RMSE, and MAE of the time-normalized and amplitude-normalized (0–1) vertical ground reaction force (vGRF). Results: The algorithm successfully synchronized 92.5% of the 330 records; the remaining cases showed kinematic noise or additional steps that prevented robust event detection. During validation, correlations were r = 0.85 (60°) and r = 0.81 (90°), with Root Mean Square Error (RMSE) < 0.17 and Mean Absolute Error (MAE) < 0.14, values representing less than 0.1% of the peak force. Conclusions: The heel-contact-based algorithm allows accurate synchronization of OpenCap and force platform signals during lower-limb functional exercises, achieving performance comparable to hardware-synchronized systems. This approach facilitates the integration of markerless motion capture in clinical, sports, and occupational settings where advanced dynamic analysis is required with limited infrastructure.

## 1. Introduction

Biomechanical analysis of functional tasks requires the precise integration of kinematic and kinetic information to reliably characterize the mechanical demands imposed on the musculoskeletal system [1]. Temporal synchronization between these signals is critical for the estimation of ground reaction forces, as well as for the validation of musculoskeletal models and for clinical and sport-related decision-making [2]. Traditionally, these analyses have been performed in laboratories equipped with optical motion capture systems and force platforms synchronized through hardware, which are considered the reference standard (“Gold Standard”). However, the complexity and limited portability of these systems restrict their routine use in clinical, rehabilitation, and applied environments outside the laboratory.

In recent years, markerless motion capture systems based on video cameras and computer vision models have emerged as a lower-cost and more accessible alternative [3]. Among these, OpenCap [4] has been established as an open-source tool that allows the estimation of three-dimensional kinematics and dynamic variables from videos acquired with smartphones, integrating musculoskeletal models and simulation workflows through software such as OpenSim 4.5 [5]. Recent studies have shown that these systems can achieve levels of accuracy comparable to traditional approaches for describing kinematics and, in some cases, ground reaction forces during functional tasks and gait, provided that appropriate calibration and processing procedures are implemented [6,7].

However, the adoption of markerless systems in combination with force platforms poses a relevant methodological challenge: temporal synchronization between independently acquired signals in the absence of a shared hardware trigger. When physical synchronization is not available, it is necessary to rely on algorithms that align signals based on observable events, such as foot contact, and that are sufficiently robust to natural movement variability, signal noise, and potential tracking losses [8]. Although the literature has explored different methods for detecting contact events from kinematics and comparing them with force platform outputs [9,10], there remains a need for open, reproducible, and specifically validated procedures to integrate OpenCap with dynamic measurement devices in complex functional tasks beyond gait [11].

From an applied perspective, the availability of a reliable synchronization method between OpenCap and force platforms would allow the extension of advanced biomechanical analysis to scenarios where infrastructure is limited but precise quantification of the mechanical response of the locomotor system is required. This includes the assessment of bodyweight functional exercises, rehabilitation programs, sport performance analysis, and ergonomic studies of occupational tasks [12,13].

In this context, the present study aimed to develop and validate an automatic synchronization algorithm based on the detection of heel kinematic events to align markerless motion capture data obtained with OpenCap with force platform signals during the execution of lower-limb functional exercises in normal-weight adult women. Specifically, it sought to: (i) evaluate the successful synchronization rate of the algorithm across a broad set of tasks, including squats, lunges, and step exercises; and (ii) quantitatively compare the quality of synchronization against a Gold Standard system (OptiTrack + hardware-synchronized force platforms) by analyzing the correlation and error between the vertical ground reaction forces obtained from both systems.

## 2. Materials and Methods

### 2.1. Experimental Setup

**Figure 1:**
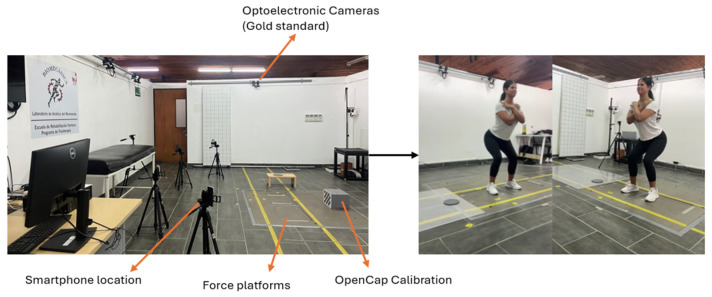
The laboratory coordinate system used by the force platforms was preserved and no axis remapping was applied.

The experimental setup consisted of an optoelectronic motion capture system used as the gold-standard reference, synchronized with force platforms and smartphone-based recordings acquired using OpenCap. Smartphones were positioned around the capture volume following the OpenCap calibration procedure, while force platforms were embedded in the laboratory floor. The analyzed task corresponded to a stationary squat, characterized by a predominantly vertical movement without horizontal displacement. Force platform signals were acquired using their internal coordinate systems, as defined by the manufacturer, and no external redefinition of spatial coordinates was performed. A photo-based schematic of the setup is provided to facilitate understanding of the laboratory configuration and improve reproducibility, see Figure 1.

### 2.2. Study Design

An observational–experimental study was conducted to analyze the synchronization between kinematic and kinetic signals during the execution of lower-limb functional tasks. Signal processing was performed using the Python 3.8 programming language, based on kinematic data obtained from the open-source OpenCap system and force data recorded with force platforms, with the aim of developing and validating an automatic temporal alignment procedure between both capture systems.

### 2.3. Participants

The sample consisted of 30 adult women classified as normal weight (BMI 18.5–24.9 kg/m^2^), aged between 18 and 45 years, with low or moderate levels of physical activity. Each participant performed two repetitions of each task without rest periods, and the execution with the best technical quality was selected for analysis according to clinical–biomechanical criteria.

### 2.4. Functional Tasks Evaluated

Seven lower-limb movements were evaluated: squats at 60° and 90° of knee flexion, step-up and step-down tasks, lateral lunge, posterior lunge, and two sliding-lunge variants (lateral and posterior), performed bilaterally when appropriate. During squats, both feet were positioned on the force platforms. In the step exercise, support was centered on the evaluated limb, placing the step on the corresponding platform. Lateral and posterior lunges were performed ensuring that the analyzed limb contacted the active platform, and in the sliding variants, the platform arrangement was adjusted to allow free sliding of the non-evaluated limb without interfering with force recording.

### 2.5. Instrumentation and Experimental Setup

A three-dimensional OptiTrack motion capture system with eight cameras and BTS P6000 force platforms was used as the reference system (“Gold Standard”). Thirty-nine reflective markers were placed over the entire body following a standardized anatomical protocol. In parallel, four iPhone 12 devices (60 fps) were positioned around the capture volume and configured with the OpenCap application for multi-camera acquisition. The OptiTrack system was managed using Motive [14], integrating a hardware trigger to synchronize cameras and force platforms, which was later used as an external reference for validating the synchronization procedure.

### 2.6. Data Acquisition and Processing

Videos acquired with OpenCap were processed in the cloud following the official platform workflow, and three-dimensional coordinates and joint variables were downloaded from the associated processing repository. Force signals from the BTS P6000 platforms were stored in the cloud, and column headers were standardized to ensure compatibility with Python scripts. For the sliding lunge tasks, platform assignment was adjusted so that the active platform matched the evaluated limb, respecting the biomechanical logic of the analysis.

### 2.7. Kinematic–Kinetic Synchronization Algorithm

Synchronization between OpenCap kinematic signals and force-plate recordings was based on the automatic detection of temporal events derived from the vertical heel trajectory and the vertical ground reaction force (vGRF). To ensure signal stability, all force signals were low-pass filtered with a 4th-order Butterworth filter at 30 Hz, while the kinematic data provided by OpenCap were filtered using the platform’s internal processing routines (cut-off frequencies between 4 and 10 Hz depending on the variable) and an additional smoothing process applied to the heel vertical coordinate. Specifically, the heel signal was normalized using min–max normalization and subsequently filtered using a cascade of two 4th-order Butterworth low-pass filters (cut-off frequency = 3 Hz, fs = 60 Hz, bidirectional application) together with a median filter with a 5-sample window to reduce residual noise.

Heel-contact detection relied on amplitude- and slope-based criteria applied to the filtered heel trajectory. A baseline reference was computed as the difference between the maximum value observed after the stabilization period and the global minimum of the signal. From this reference, an adaptive threshold was defined as:

threshold = baseline × threshold_factor + minimum signal value,

with threshold_factor set to 0.6 in single-leg tasks and 0.2 in bilateral tasks. Heel contact was detected when the ascending portion of the heel signal exceeded this threshold following a local minimum.

In parallel, force-signal onset was identified from the vGRF curves. The baseline force level was defined as the mean force of the initial resting segment (first 10 samples), and the activation threshold was computed as:

threshold = baseline + SD (signal) × threshold_factor,

with threshold_factor = 0.1. The first sample exceeding this adaptive threshold was taken as the onset of vertical loading.

**Figure 2:**
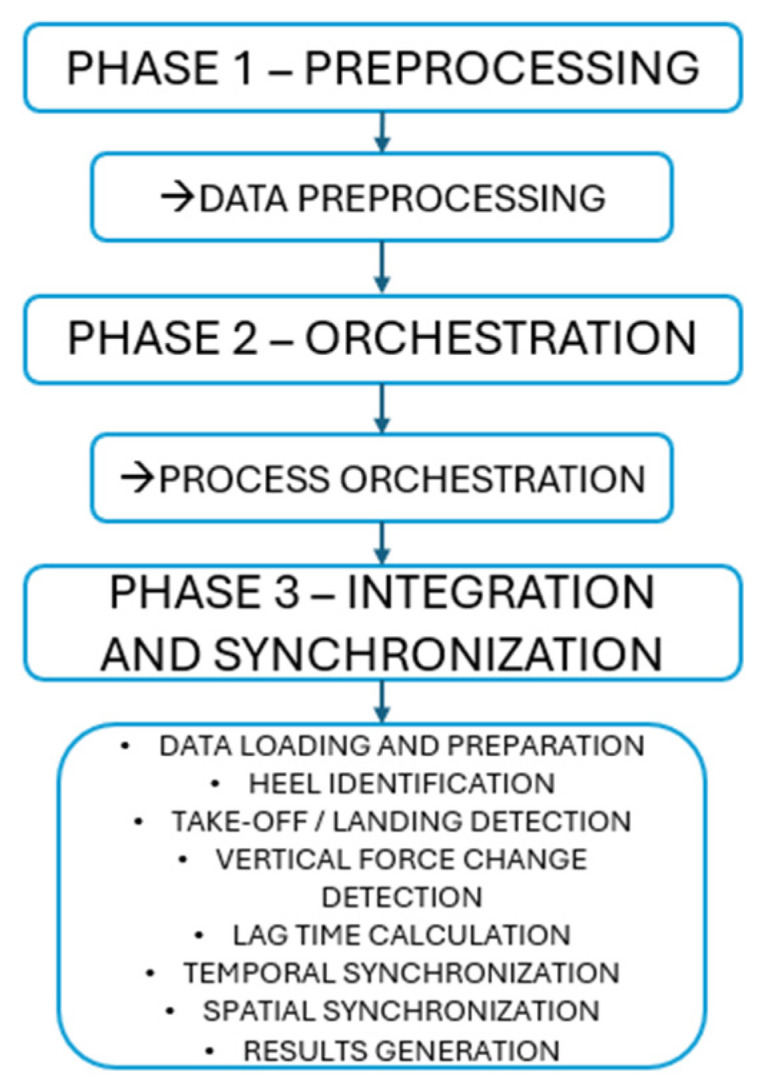
Flowchart of the synchronization process.

The temporal offset between the estimated kinematic heel-contact instant and the force onset event was then calculated and used to shift the force signal in time. After correction, all signals were expressed on a normalized temporal scale from 0 to 100% of the movement cycle. Full details of the implementation and processing routines are documented in the analysis repository (Supplementary Materials), see Figure 2.

### 2.8. Validation of the Synchronization Procedure

For validation purposes, vertical ground reaction forces from both systems were amplitude-normalized to a 0–1 range and time-normalized to 0–100% of the movement cycle to enable direct waveform comparison independent of absolute magnitude. Therefore, the reported RMSE and MAE values are dimensionless and correspond to differences in normalized force profiles rather than absolute Newton values.

The accuracy of the synchronization procedure was evaluated in a specific test with one female participant (28 years old, 62 kg) who performed squats at 60° and 90° of knee flexion (10 repetitions per condition, 110.7 s of total movement). For each repetition, the normalized vertical forces obtained from the Gold Standard system were compared with those derived from OpenCap processing after synchronization. Both signals were aligned using the same kinematic event (first heel contact) and expressed as a percentage of the movement cycle. The objective of this phase was to validate the temporal accuracy of the synchronization algorithm, not the inter-individual variability of reaction forces. Synchronization is a mathematical-computational process whose accuracy depends on the detection of kinematic events, not on individual anthropometric characteristics. The 20 repetitions provided sufficient intra-subject variability in terms of execution velocity, flexion depth, and muscle activation patterns, allowing evaluation of the algorithm’s robustness to natural movement variations. This approach is consistent with previous studies validating synchronization and event detection algorithms [15,16,17], where temporal precision is established through detailed analysis of multiple cycles under controlled conditions before being applied to broader populations.

## 3. Results

A total of 330 motion records corresponding to the 30 participants were analyzed, who performed between 11 and 14 lower-limb functional tasks. The proposed synchronization system successfully aligned 92.5% of the trials. The remaining 7.5% could not be processed due to kinematic noise, temporary tracking losses, or the execution of additional steps before stepping onto the force platform.

The quality of synchronization was evaluated by the temporal offset between the estimated heel-contact instant and the initial rise in the vertical force. The overall mean offset was −1.38 s (SD = 0.73 s), where negative values indicate that the force signal was activated before the estimated kinematic instant. Table 1 presents the means and standard deviations by movement type. In general, values ranged from −1.04 s to −1.53 s, showing a consistent trend across tasks.

**Figure 3:**
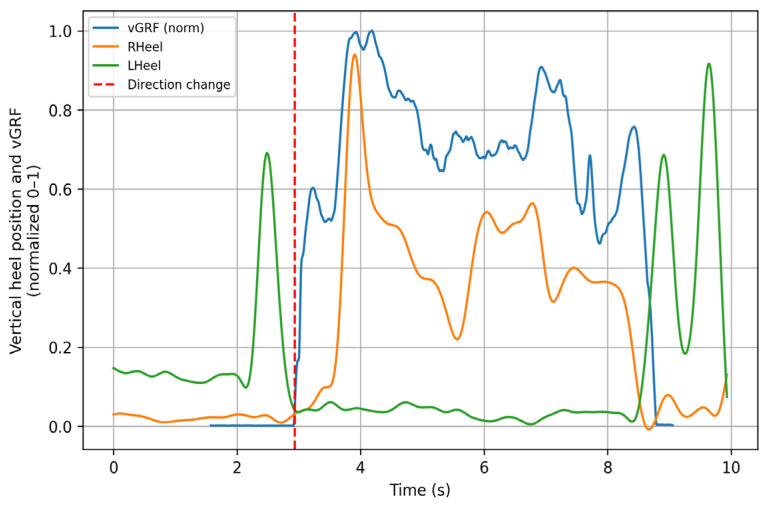
Left posterior sliding lunge. The green trace (LHeel) corresponds to the normalized vertical movement of the left heel. The red dashed line (change of direction) indicates the instant when the heel contacts the ground. The blue trace (vGRF) represents the normalized vertical ground reaction force recorded by the force plates, and the orange trace (RHeel) corresponds to the normalized vertical movement of the right heel. All signals are normalized between 0 and 1. *Y*–axis: Vertical magnitude (normalized 0–1). *X*–axis: Time (s).

Figure 3 illustrates the performance of the algorithm during the left posterior sliding lunge, showing how the force signal is temporally shifted to match the instant of support of the evaluated limb (red dashed line). This behavior was consistently replicated across most of the analyzed movements. The figure shows both the heel trajectory and the force platform signals; for visualization purposes, all signals were normalized. This graph is intended for demonstrative purposes, highlighting how the algorithm produces a fully synchronized signal.

**Figure 4:**
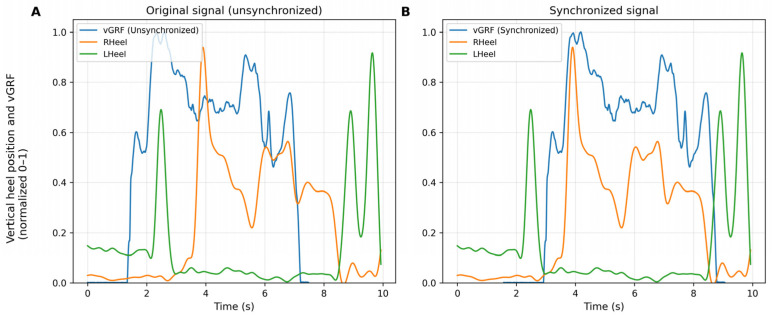
Synchronized vs. unsynchronized data. (**A**) Original signal before applying the synchronization algorithm. (**B**) Signal after synchronization. The blue trace corresponds to the vertical ground reaction force (vGRF), while the orange and green traces represent the vertical position of the right and left heel, respectively. All signals are normalized between 0 and 1 for direct comparison. *Y*–axis: Vertical magnitude (normalized 0–1). *X*–axis: Time (s).

Figure 4 shows the comparison between the original (unsynchronized) signals and the synchronized signals after applying the algorithm. A clear alignment is observed between the vertical force and the heel kinematics, especially during the initial contact phase, confirming the effectiveness of the temporal adjustment.

**Figure 5:**
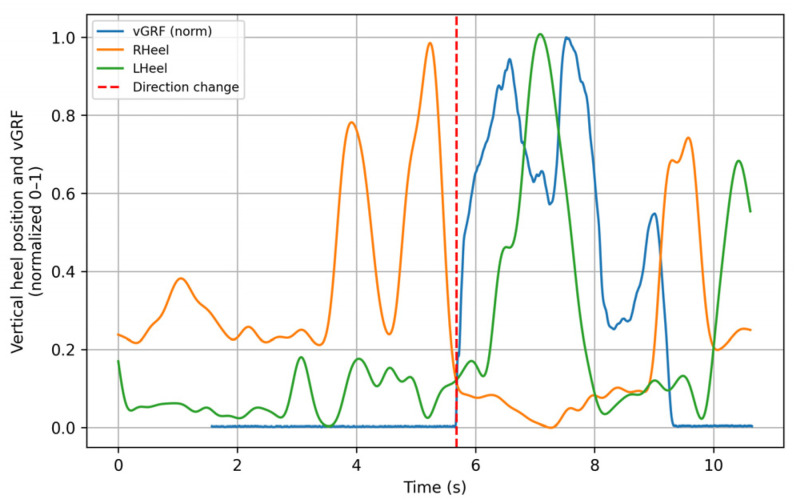
Right posterior sliding lunge showing an additional step prior to the target foot contact.

In some cases, atypical patterns were observed, such as additional steps before the main movement. Figure 5 illustrates this situation in a right posterior sliding lunge, where the participant performed a preliminary step that generated a force peak not corresponding to the target movement contact. In these cases, manual signal adjustment was required because the algorithm detected the contact prematurely.

### 3.1. Validation Against the Gold Standard System

The algorithm was validated by comparing the normalized vertical forces obtained from the Gold Standard system with those generated by OpenCap after synchronization. The test was applied to one female participant (28 years old, 62 kg) during 20 squat repetitions (10 at 60° and 10 at 90°), corresponding to 110.7 s of total movement.

Signals from both systems were aligned using the same kinematic event (first heel contact) and rescaled to 100% of the movement cycle. Pearson correlation (r), RMSE, and MAE were calculated, and the results are presented in Table 2.

The correlation values were r = 0.85 ± 0.12 for the 60° squat and r = 0.82 ± 0.28 for the 90° squat, which are considered indicators of excellent synchronization (r > 0.80). In 15 of the 20 trials (88%), correlations above 0.80 were obtained. Trial 90_6 showed a low correlation (r = 0.23) attributable to a transient tracking loss in OpenCap, not associated with the performance of the algorithm.

Absolute errors were low: RMSE < 0.17 and MAE < 0.14, which correspond to less than 10% of the full normalized force range, while relative differences between conditions were small (−4.3% in correlation, −5.9% in RMSE, and −30.3% in MAE). These findings indicate that the synchronization method produces signals comparable to those of the reference system in terms of waveform shape and temporal alignment, with sufficient accuracy for biomechanical analysis applications.

### 3.2. Temporal Validation of the Algorithm (Direct Curve Comparison)

In addition to quantifying the raw temporal offset, the temporal agreement between the kinematic and kinetic signals after synchronization was evaluated. Figure 4 and Figure 5 show the mean and variability (±1 SD) of the vertical heel position and the normalized vertical force for the 60° and 90° squat repetitions, respectively. The superposition of both signals shows that the algorithm adequately aligns the contact events and the main phases of the movement cycle, with discrepancies observed only in magnitude, which are attributable to the markerless system.

**Figure 6:**
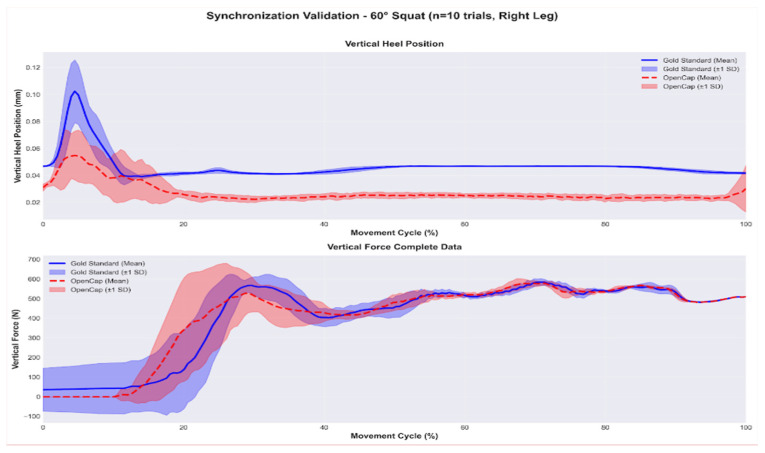
Averaged curves of heel position and synchronized vertical force—60° squat (OpenCap—red, Gold Standard—blue).

**Figure 7:**
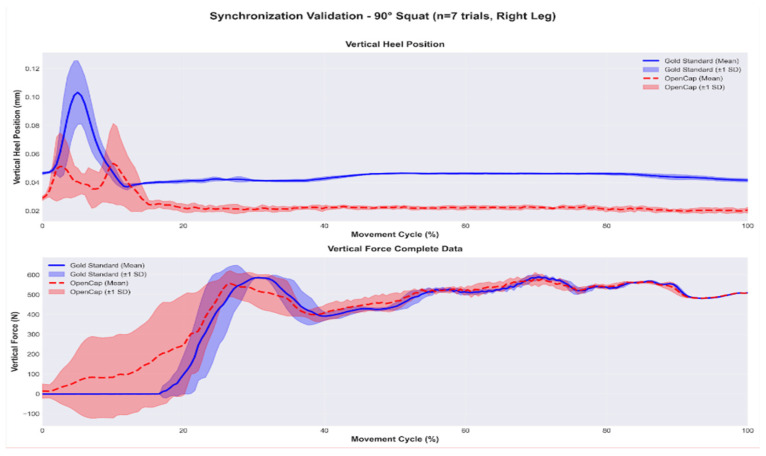
Averaged curves of heel position and synchronized vertical force—90° squat (OpenCap—red, Gold Standard—blue).

Figure 6 and Figure 7 complement these findings by showing the point-by-point relationship between the vertical forces recorded by the Gold Standard and those estimated by OpenCap. In each figure, the left panel includes all data points from the trials, while the right panel presents a close-up based on kernel density estimation (KDE), highlighting the region of greatest consistency between both systems.

**Figure 8:**
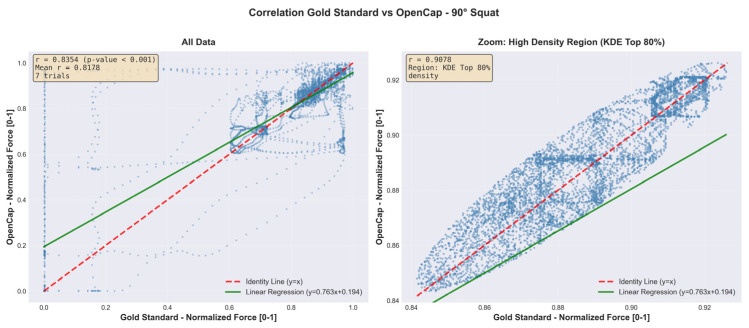
Scatter plot with KDE zoom (OpenCap vs. Gold Standard agreement)—90° squat.

**Figure 9:**
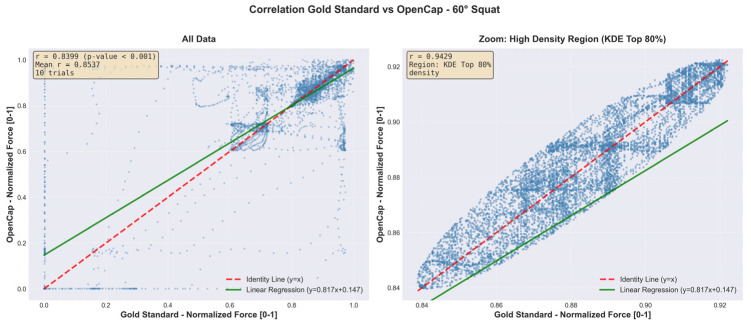
Scatter plot with KDE zoom (OpenCap vs. Gold Standard agreement)—60° squat.

This analysis demonstrates a high level of agreement during the stable phase of the cycle, with correlations greater than 0.90 within the highest-density cluster. Overall, these results indicate that, once synchronized, the vertical forces estimated by OpenCap adequately reproduce the temporal pattern and the relative magnitude of the signals obtained with the reference system, see Figure 8 and Figure 9.

## 4. Discussion

The proposed algorithm, based on the automatic detection of heel contact, demonstrated accurate temporal alignment between the kinematics estimated by OpenCap and the kinetic signals from the force platforms. The successful synchronization rate (92.5%) and the high correlations against the Gold Standard system (r > 0.80 in 88% of the trials) indicate that this approach is reliable and robust, even in the presence of marked variations in movement patterns.

### 4.1. Interpretation of the Findings

The correlations obtained (r = 0.85 for 60° and r = 0.81 for 90°) show that the algorithm maintains stable accuracy despite changes in squat depth. The relative difference between both conditions (−4.3%) is small and suggests that the method does not strictly depend on a specific kinematic configuration, but rather adapts to natural variations in execution.

This stability is clearly reflected in Figure 4 and Figure 5, which present the averaged curves of the vertical heel position and the synchronized vertical force for 10 trials at 60° and 7 trials at 90°. In both conditions, the synchronized signals show consistent temporal overlap between OpenCap and the Gold Standard, indicating that the algorithm correctly identifies contact events, the onset of the loading phase, and the final ascent of the movement. Although differences in magnitude persist between systems, the coincidence of peaks, transitions, and global patterns confirms that the synchronization does not introduce artificial temporal shifts and fulfills its primary objective of ensuring temporal compatibility between kinematics and force. This aspect is particularly relevant in tasks with variable ranges of motion, where force dynamics critically depend on the instant of contact.

These values fall within the range reported in validation studies of markerless systems and in comparisons between motion capture systems [18]. Uhlrich et al. (2023) [5] reported correlations above 0.80 when comparing OpenCap with optoelectronic systems and force platforms, while Bonakdar et al. (2025) [19] and the methodological approaches proposed by Bland and Altman (1986) [20] described agreements between 0.80 and 0.95 across different measurement configurations. Complementarily, D’Haene et al. (2024) [21], Valenzuela et al. (2025) [22], and Horsak et al. (2025) [23] showed that kinematics and dynamic behavior estimated with markerless systems can faithfully reproduce both locomotion tasks and simulated pathological conditions. In this context, the average correlation observed in the present study (r ≈ 0.85) suggests that the performance of the algorithm is comparable to that of hardware-based synchronization procedures, but with lower instrumental complexity.

The point-by-point agreement analysis (Figure 6 and Figure 7) reinforces this interpretation. The full panels show a positive global relationship between OpenCap and Gold Standard estimates, while the kernel density estimation (KDE) zoomed panels reveal a high-consistency cluster during the stable phase of the cycle, with correlations greater than 0.90. This result indicates that, once synchronized, the vertical forces estimated by OpenCap reliably reproduce both the temporal evolution and the relative magnitude of the reference signals, particularly during sustained loading periods, which are critical for biomechanical analysis.

Taken together, Figure 4, Figure 5, Figure 6 and Figure 7 demonstrate that the algorithm not only corrects the initial temporal offset between systems, but also consistently preserves the biomechanical relationship between movement and force throughout the full movement cycle. These results support its usefulness for advanced kinetic analyses, including the estimation of joint contact forces and the validation of musculoskeletal models.

### 4.2. Strengths of the Algorithm

#### 4.2.1. Stability Under Kinematic Variability

The similarity of the results between 60° and 90° squats indicates that the algorithm generalizes well across different movement depths, a property that is especially relevant in clinical and sports contexts where execution presents inter-subject variability. Similar findings have been reported by Yang (2024) [24] and Verheul et al. (2024) [25] who observed stable temporal accuracy of markerless systems in tasks with varying movement amplitudes, reinforcing the idea that synchronization based on kinematic events can remain stable despite changes in movement strategy.

#### 4.2.2. Temporal Accuracy and Error Magnitude

The absolute errors obtained (RMSE < 0.17; MAE < 0.14) represent less than 0.1% of the maximum force peak and are consistent with recent studies using markerless kinematics to estimate ground reaction forces. Feng et al. (2025) [26] highlighted that, after careful event alignment, errors can fall below 1% of the maximum magnitude, in line with the observations of the present study. The combination of high correlations and low-magnitude errors supports the use of the algorithm for fine dynamic analyses in environments where temporal precision is critical.

#### 4.2.3. Reliability of the Kinematic Event

The use of heel contact as the reference synchronization event aligns with evidence from Patoz et al. (2021) [27] and Zahradka et al. (2020) [15], who demonstrated that the detection of foot contact events from kinematics shows high agreement with force platform recordings. The strategy based on relative thresholding and ascending slope therefore emerges as a viable alternative to solutions dependent on external triggers or additional sensors, while maintaining low cost and less intrusive configurations.

### 4.3. Limitations

The quality of tracking directly influences the performance of the algorithm; marker loss or excessive noise may compromise event detection, as observed in the small percentage of trials that required manual intervention [28]. In addition, the discrepancy in sampling frequency between OpenCap (60 Hz) and the force platforms (1000 Hz) may smooth the synchronized force response and attenuate rapid peaks. The incorporation of spline interpolation techniques and adaptive filtering could mitigate these effects in future versions of the procedure. Finally, the validation against the Gold Standard system was conducted with one participant performing a single task type (squats at two knee flexion angles). While this approach is consistent with algorithm validation studies focused on temporal accuracy [15,16,17], it limits generalizability across diverse populations and movement tasks.

### 4.4. Implications and Applications

The results of this study show that an event-based synchronization algorithm enables the reliable integration of a low-cost markerless system (OpenCap) with laboratory force platforms during lower-limb functional tasks. The high proportion of correctly synchronized trials (92.5% of 330 records) and the strong correlations with the reference system (r > 0.80 in 88% of squat repetitions) indicate that the achieved accuracy is sufficient for advanced kinetic analysis applications, including the assessment of ground reaction forces and the validation of musculoskeletal models.

The fact that the algorithm maintained stable performance across movements with different mechanical demands—60° and 90° squats, lunges, sliding variants, and step tasks—suggests that it can be used in clinical, sports, and occupational contexts where execution presents inter-subject variability and where both bilateral and unilateral support phases are combined. In addition, the method’s ability to automatically identify problematic records, such as those with additional steps or kinetic peaks not associated with the target movement, is particularly useful in rehabilitation or functional assessment scenarios, where movement strategies tend to be less regular. Taken together, these findings expand the potential use of OpenCap as an accessible tool for biomechanical studies that require precise synchronization with dynamic measurements without relying on hardware-based synchronization infrastructure.

### 4.5. Future Perspectives

Although the detailed validation against the Gold Standard system was performed in a single participant for the squat exercise, the complete cohort of 30 women and the 330 analyzed repetitions provide a natural framework for extending the analysis at the population level. Future studies should explore how anthropometric variables, execution strategies, and motor control levels affect synchronization accuracy, especially in more complex tasks such as sliding lunges or movements that include preparatory steps.

In addition, the discrepancy in sampling frequency between OpenCap (60 Hz) and the force platforms (1000 Hz) highlights the need to investigate interpolation and resampling techniques that allow more precise alignment of force peaks in high-impact or short-duration movements. The incorporation of machine learning approaches for adaptive event detection could improve the algorithm’s performance under conditions of high noise or partial tracking loss, reducing the need for manual adjustments. Finally, integrating this synchronization procedure into musculoskeletal simulation workflows based on OpenCap opens the possibility of more rigorously evaluating joint and muscle forces, which would be particularly valuable in clinical, ergonomic, and biomechanical risk assessment studies.

Future research should prioritize systematic validation of the synchronization algorithm across multiple participants with diverse anthropometric characteristics, training levels, and movement patterns, including clinical populations with pathological gait or movement disorders. Validation should also extend to varied movement tasks (jumping, running, cutting maneuvers) and ecologically valid environments beyond controlled laboratory settings. Expanding the analysis to include all three components of ground reaction forces (anterior–posterior and mediolateral in addition to vertical) and implementing automated quality control metrics for real-time error detection would enhance the system’s clinical applicability. Longitudinal studies examining test–retest reliability and sensitivity to clinically meaningful changes are necessary to establish this technology as a viable tool for rehabilitation monitoring and return-to-sport decision-making.

## 5. Conclusions

This study developed and validated an automatic synchronization algorithm based on heel contact to align markerless motion capture data (OpenCap) with force platform signals during lower-limb functional exercises. The algorithm successfully synchronized 92.5% of the 330 records and showed high correlations and very low errors compared with a Gold Standard system, demonstrating adequate temporal accuracy for advanced kinetic analyses.

The results indicate that the proposed method represents a viable alternative for integrating OpenCap with force platforms in clinical, sports, and occupational environments with limited infrastructure, and opens the possibility of expanding the use of markerless systems in biomechanical studies that require reliable dynamic information.

## Figures and Tables

**Table 1:** Pre-synchronization temporal offset between kinematic heel-contact and force-plate signal onset. Negative values indicate that the force signal occurred earlier than the heel-contact event estimated from OpenCap kinematics. Values correspond to the mean and standard deviation of the temporal offset (in seconds) for each functional task before applying the synchronization algorithm.

Movement	Mean (s)	Standard Deviation (SD, s)
Squat 60°	−0.959531	0.876380
Squat 90°	−0.835037	1.293524
Right step-up/step-down	−1.481986	0.610084
Left step-up/step-down	−1.522078	0.479812
Right lateral lunge	−0.832069	0.810606
Left lateral lunge	−0.722510	1.127811
Right posterior lunge	−1.253630	1.020298
Left posterior lunge	−1.435783	0.518344
Right lateral sliding lunge	−1.334426	0.568691
Left lateral sliding lunge	−1.336467	0.544159
Right posterior sliding lunge	−1.378844	1.002620
Left posterior sliding lunge	−1.676133	0.438092

**Table 2:** Validation metrics between systems.

Parameter	Squat 60°	Squat 90°	Difference
Correlation (r)	0.8537 ± 0.1165	0.8178 ± 0.2846	−4.3%
RMSE	0.17 ± 0.07	0.16 ± 0.12	−5.9%
MAE	0.14 ± 0.07	0.10 ± 0.09	−30.3%

## Data Availability

The original contributions presented in this study are included in the article/Supplementary Materials. Further inquiries can be directed to the corresponding author.

## Supplementary Materials

The following supporting information can be downloaded at: https://www.mdpi.com/article/10.3390/s26020360/s1 and https://github.com/Diego-AArturo/opencap-forceplate-sync (accessed on 22 December 2025).
