# Supplementary material for: Synchronization of OpenCap with Force Platforms: Validation of an Event-Based Algorithm
Source: Sensors (Basel). 2026 Jan 6;26(2):360. doi: 10.3390/s26020360 (PMC12846238; doi:10.3390/s26020360)
Supplement: Supplementary file 1 [file sensors-26-00360-s001.zip › sensors-4053056-supplementary.pdf]

Integration of Force Platforms with OpenCap

Overview

This project provides a set of tools designed to synchronize force platform data with kinematic data obtained using OpenCap. Its primary objective is to accurately integrate the temporal and spatial measurements from force platforms with the markerless motion-capture system of OpenCap, enabling comprehensive biomechanical analyses.

Project repository: https://github.com/Diego-AArturo/opencap-forceplate-sync

This project is based on and extends the functionalities of the original repository:

Original repository: https://github.com/stanfordnmbl/opencap-processing
Developed by: Stanford Neuromuscular Biomechanics Laboratory

We thank the Stanford Neuromuscular Biomechanics Laboratory for providing the foundational OpenCap processing tools that made this project possible.

Main Objective

The main objective of this system is to synchronize OpenCap kinematic data with force platform measurements, allowing researchers to combine:

- **Kinematic data from OpenCap** (marker positions, joint angles, etc.)
- **Kinetic data from force platforms** (ground reaction forces, moments, center of pressure)

This synchronization enables comprehensive biomechanical analyses, including inverse dynamics calculations in OpenSim.

Quick Start

The main script to run is `scripts/batch_process_forceplates.py`, which automatically processes multiple participants and movements.

Step-by-Step Workflow

Step 1: Preprocess Force Platform Files
Before processing, the force platform files must be preprocessed using the Jupyter notebook:

`notebooks/plataformas_fuerza.ipynb`

This notebook:

- Processes raw force platform data files
- Standardizes column naming conventions
- Handles special cases (e.g., lateral sliding movements require swapping leg labels)
- Prepares files for integration

Important: For lateral sliding movements (`deslizamiento_lateral`), the system automatically swaps right/left leg labels `(r_ ↔ L_, l_ ↔ R_)` to match the movement pattern.

Step 2: Create Participant Configuration JSON File

After preprocessing, create a JSON file that links the force platform data with the OpenCap video sessions. The movement names in the JSON must match the test names in OpenCap **exactly**.

File Structure (example: `participantes.json`):

```
[

  {

    "participant_id": "P1",

    "session_id": "opencap_session_id_aqui",

    "movements": [

      {

        "move": "nombre_movimiento",

        "link": "url_google_drive_o_vacio"

      },

      {

        "move": "nombre_movimiento_2",

        "link": "url_google_drive_o_vacio"
```

```
          }
        ]
    },
    {
        "participant_id": "P2",
        "session_id": "otro_opencap_session_id",
        "movements": [
          {
              "move": "nombre_movimiento",
              "link": "url_google_drive_o_vacio"
          }
        ]
    }
]
```

Key Requirements:

- `participant_id:` Unique identifier for the participant
- `session_id:` OpenCap session ID (found in the OpenCap web interface)
- `move:` Must match the test name in OpenCap exactly
- `link:` Google Drive URL for force platform data (may be left empty if using local files)

Step 3: Configure and Run Batch Processing

1. Edit `scripts/batch_process_forceplates.py`:

- Update the variable `name_file` to match the name of your JSON file (without the `.json` extension)
- Configure the movement classification sets (`unprocessed, bothlegs`) if needed

2. Run the script:

   python scripts/batch_process_forceplates.py

3. script:

   - It will load participant data from the JSON file
   - It will download or load force platform data
   - It will download OpenCap kinematic data (if not already available)
   - It will synchronize temporal and spatial coordinates
   - It will run inverse dynamics analyses in OpenSim
   - It will generate synchronization plots

Step 4: Access Synchronized Data

After processing, the synchronized data can be found in:

```
Data/
└── {participant_id}/
    ├── MeasuredForces/
    │   └── {trial_name}/
    │       └── {trial_name}_syncd_forces.mot
    ├── OpenSimData/
    │   ├── InverseDynamics/
    │   │   └── {trial_name}/
    │   └── Kinematics/
    └── MarkerData/
```

Integration Methods

The system provides two integration methods depending on the type of movement and the available information:

Method 1: Single-Leg Integration

File: `src/forceplates/funtion_integrate_forceplates_legs.py`
Function: `IntegrateForcepalte_legs()`

Use when:

- Only one foot contacts the force platform
- The contacting foot is known
- The movement is clearly unilateral (e.g., single-step, unilateral lunge)

Parameters:

- `legs:` Specify `'R'` for right leg or `'L'` for left leg

Example:

```
IntegrateForcepalte_legs(

    session_id=session_id,

    trial_name="escalon_derecho_1",

    force_gdrive_url=force_url,

    participant_id="P1",

    legs='R'  # Especifique la pierna

)
```

2: Both-Legs Integration (Auto-Detection)

File: `src/forceplates/integrate_forceplates_both_legs.py`
Function: `IntegrateForcepalte_vc0()`

Use when:

- Both feet contact the force platforms simultaneously
- It is not known which foot contacted first
- The movement is bilateral (e.g., squats, jumps, bilateral lunges)

Features:

- Automatically detects which leg initiated contact first

- Compares heel-contact events from both legs
- Uses combined force signals for synchronization

Example:

IntegrateForcepalte_vc0(

session_id=session_id,

trial_name="sentadilla_90_1",

force_gdrive_url=force_url,

participant_id="P1"

No leg parameter is needed — it is detected automatically

)

Automatic Selection:

The batch processing script (`batch_process_forceplates.py`) automatically selects the appropriate method based on movement name patterns:

- Movements containing `'derecha'` or `'derecho'` → Single-leg (right)
- Movements containing `'izquierda'` or `'izquierdo'` → Single-leg (left)
- Movements in the `bothlegs` set (e.g., `'sentadilla'`) → Both-legs integration

Validation with Gold Standard

To validate the synchronization results against a gold-standard system (e.g., Motive motion capture with hardware-synchronized force platforms), use the validation scripts located in the `src/validation/ folder.`

Quick Validation Script

File: `src/validation/autocode4val.py`

script processes multiple trials and compares synchronized OpenCap data with gold-standard measurements.

Comprehensive Validation

File: `src/validation/validation_sync.py`

The `SyncValidator` class provides comprehensive validation including:

- Comparison of temporal alignment
- Correlation analysis of force signals
- RMSE and MAE calculations
- Visualization plots
- Detailed validation reports

Usage Example:

```
from src.validation.validation_sync import SyncValidator

validator = SyncValidator(

    gold_standard_mot_path="ruta/a/gold_forces.mot",

    gold_standard_trc_path="ruta/a/gold_markers.trc",

    opencap_syncd_mot_path="Data/P1/MeasuredForces/trial1/trial1_syncd_forces.mot",

    opencap_trc_path="Data/P1/MarkerData/trial1.trc",

    output_folder="validation_results"

)

metrics = validator.run_validation()
```

Output Files

Synchronized Force Data

- **Location:** `Data/{participant_id}/MeasuredForces/{trial_name}/{trial_name}_syncd_forces.mot`
- **Format:** OpenSim MOT file containing synchronized force platform data
- **Content:** Ground reaction forces, moments, and center of pressure for both legs

Inverse Dynamics Results

- **Location:** `Data/{participant_id}/OpenSimData/InverseDynamics/{trial_name}/{trial_name}.sto`
- **Format:** OpenSim STO file
- **Content:** Joint moments and powers computed from synchronized data

Synchronization Plots

- **Location:** `graficas/{participant_id}/{trial_name}_corte.png`
- **Content:** Visualization of heel position and synchronization point

Lag Times

- **File**: `lag_times.json`
- **Content:** Temporal shift values calculated for each trial
- **Purpose:** Records synchronization parameters for reference

Project Structure

```
ForcePlateIntegration/
├── scripts/
│   └── batch_process_forceplates.py      # Main batch processing script
├── src/
│   ├── forceplates/
│   │   ├── funtion_integrate_forceplates_legs.py    # Single-leg integration
│   │   └── integrate_forceplates_both_legs.py      # Both-legs integration
│   └── validation/
│       ├── validation_sync.py           # Validation
│       └── autocode4val.py              # Quick validation script
├── notebooks/
```

```
│   └── plataformas_fuerza.ipynb        # Force platform preprocessing
├── Data/                               # Output directory
└── README_es.md                        # File
```

Requirements

Refer to `requirements.txt` for the full list of dependencies. Key dependencies include:

- numpy
- pandas
- matplotlib
- scipy
- opensim
- requests

Notes

- Movement names in the JSON configuration must match OpenCap test names exactly
- Force platform files must be preprocessed before integration
- The system automatically handles coordinate system transformations
- Spatial calibration aligns force vectors with anatomical markers
- Temporal synchronization uses heel-contact detection and cross-correlation

Credits and Acknowledgments

This project is based on the OpenCap processing repository developed by the Stanford Neuromuscular Biomechanics Laboratory:

- **Original repository:** https://github.com/stanfordnmbl/opencap-processing
- Stanford Neuromuscular Biomechanics Laboratory

Repository:: https://github.com/Diego-AArturo/opencap-forceplate-sync
